# Retraction: The Nexus between VEGF and NFκB Orchestrates a Hypoxia-Independent Neovasculogenesis

**DOI:** 10.1371/journal.pone.0227432

**Published:** 2019-12-30

**Authors:** The PLOS ONE Editors

After this article [1] was published, concerns were raised about results reported in Figures 4 and 9.

Specifically:

- Given the brightness/contrast levels of the western blot images and the vertical lines separating lanes, one cannot confirm the integrity of the images or clarify whether the lanes for each panel present data from the same blot and exposure. When levels are adjusted for Figure 9, it appears as though each band represents an isolated blot fragment overlaid on a white background.
- In Figure 4C, the following bands appear similar:
  - Nuclear NFκB/p65 lane 2 (VEGF) and β-Actin lane 3 (VEGF/DMSO)
  - Nuclear NFκB/p65 lane 3 (VEGF/DMSO) and β-Actin lane 5 (VEGF/SN50)
  - Nuclear NFκB/p65 lane 4 (VEGF/SN50M) and β-Actin lane 4 (VEGF/SN50M)
- In Figure 9B, the following bands appear similar:
  - NFκB/p65 lane 2 (VEGF-Treated), SDF-1α lane 3 (DMSO-Treated), CXCR4 lane 2 (VEGF-Treated), FAK lane 2 (VEGF-Treated)
  - SDF-1α lane 2 (VEGF-Treated) and NFκB/p65 lane 3 (DMSO-Treated), when flipped vertically
  - CXCR4 lane 3 (DMSO-Treated) and FAK lane 3 (DMSO-Treated)
  - EPO lane 4 (YC-1-Treated) and ET-1 lane 3 (DMSO-Treated)

The authors were unable to resolve these issues or provide the original data underlying the results in response to journal queries. Without the original data we cannot resolve the above concerns which call into question the integrity of the images and the validity of the reported results.

In light of these concerns, the *PLOS ONE* Editors retract this article.

Per FHAM and FAAM, the underlying data for this study were held by MD for whom current contact information is not available. The journal has not received confirmation that MD received communications about this case.

FHAM and FAAM replied to our notification but did not specify agreement or disagreement with the retraction. The other authors either could not be reached or did not reply.
